# Supplementary material for: Artery formation in the intestinal wall and mesentery by intestine-derived Esm1+ endothelial cells
Source: Nat Commun. 2025 Sep 25;16:8423. doi: 10.1038/s41467-025-64047-0 (PMC12462501; doi:10.1038/s41467-025-64047-0)
Supplement: Supplementary file 1 — Supplementary Information [file 41467_2025_64047_MOESM1_ESM.pdf]

Supplementary Information

Artery formation in the intestinal wall and mesentery by intestine-derived Esm1+ endothelial cells

Esther Bovay[1], Kai Kruse[2], Emma C. Watson[1], Vishal Mohanakrishnan[1], Martin Stehling[3], Frank Berkenfeld[1], Mara E. Pitulescu[4], Mark L. Kahn[5] and Ralf H. Adams[1#]

[1]Max Planck Institute for Molecular Biomedicine, Department of Tissue Morphogenesis, D-48149 Münster, Germany.

[2]Max Planck Institute for Molecular Biomedicine, Bioinformatics Service Unit, D-48149, Münster, Germany.

[3]Max Planck Institute for Molecular Biomedicine, Flow Cytometry Unit, D-48149, Münster, Germany.

[4]Max Planck Institute for Molecular Biomedicine, Vascular Patterning Dynamics Group, D-48149 Münster, Germany.

[5]Cardiovascular Institute, Department of Medicine, Perelman School of Medicine, University of Pennsylvania, Philadelphia, Pennsylvania.

Keywords: Endothelial cells, intestine, mesentery, artery development, single-cell RNA sequencing.

Conflict of interests: authors declare no conflict of interests

[#]Author for correspondence:

Ralf H. Adams
Department of Tissue Morphogenesis
Max-Planck-Institute for Molecular Biomedicine and University of Münster
D–48149 Münster, Germany
ralf.adams@mpi-muenster.mpg.de
Phone: +49 251 70365 410; Fax: +49 251 70365 499

Supplementary Figure 1

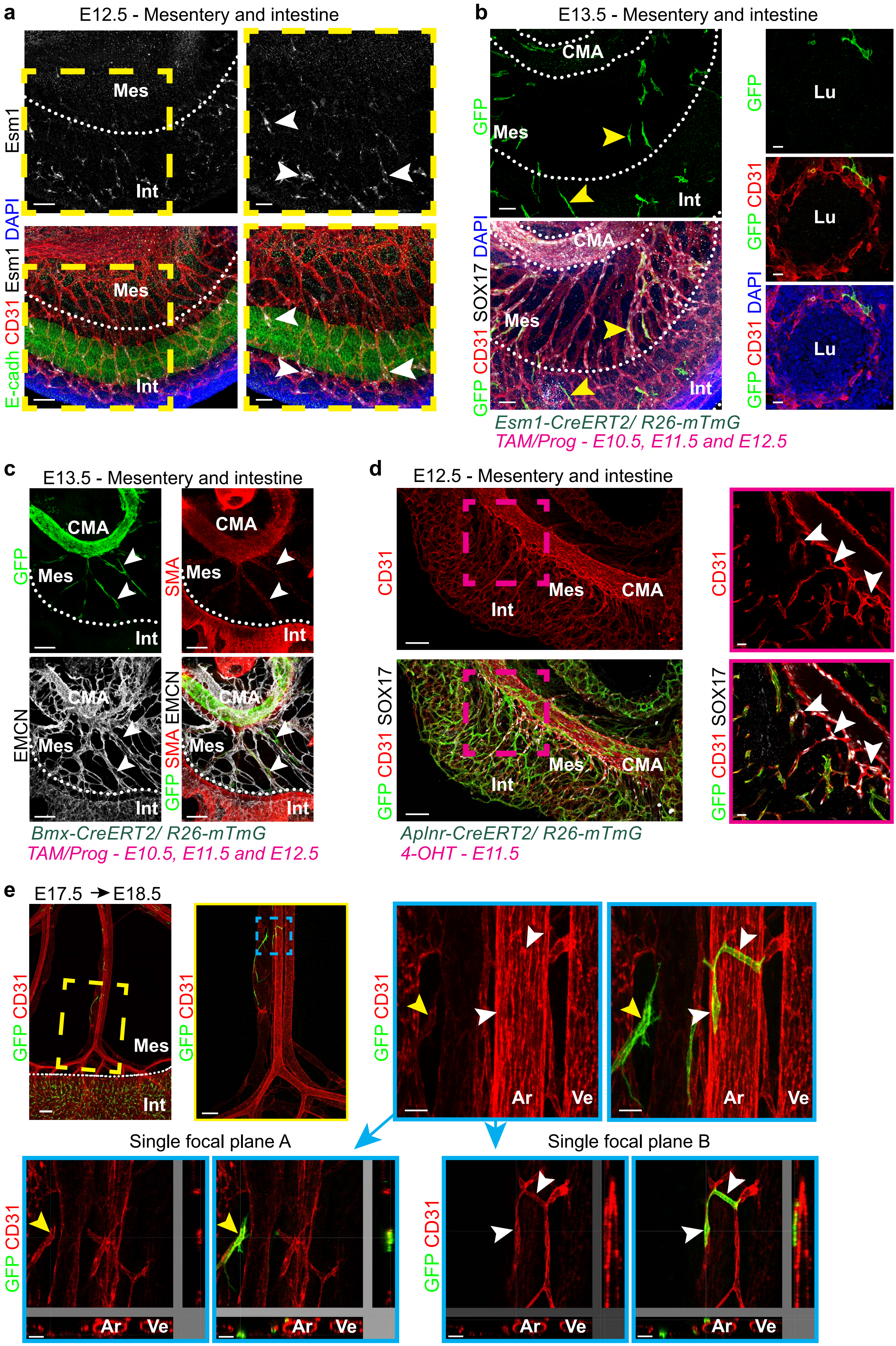

Supplementary Figure 1. Esm1^{+} cells contribute to SOX17^{+} mesenteric vessels before intestinal stratification.

(**a**) Whole-mounts of E12.5 mesentery and intestine stained for Esm1 (white arrowheads), CD31 (red), E-cadherin (green) and DAPI (blue). Mesentery (Mes) and intestine (Int) are indicated. *n* = 3. Scale bars, 50 and 30 µm. (**b**) Whole-mounts of E13.5 mesentery and intestine after daily tamoxifen treatment from E10.5 showing *Esm1-CreERT2*-labelled GFP^{+} ECs (green, arrowheads) in the SOX17^{+} mesenteric vasculature. CD31 (red), SOX17 (white) and DAPI (blue). Cranial mesenteric artery (CMA), mesentery (Mes), intestinal lumen (Lu), and intestine (Int) are indicated. *n* = 3. Scale bar, 50 µm. (**c**) Whole-mount of E13.5 mesentery and intestine after daily tamoxifen treatment from E10.5 showing *Bmx-CreERT2*-labelled GFP^{+} (green, arrowheads) ECs in the EMCN^{-} mesenteric vasculature. SMA (red) and EMCN (white). Cranial mesenteric artery (CMA), mesentery (Mes) and intestine (Int) are indicated. *n* = 4. Scale bar, 150 µm. (**d**) At E12.5, mesenteric SOX17^{+} capillaries connected to the CMA are Aplnr^{low} (arrowheads). *Aplnr-CreERT2* lineage tracing for 24h. GFP (green), SOX17 (white) and CD31 (red). Cranial mesenteric artery (CMA), mesentery (Mes) and intestine (Int) are indicated. *n* = 5. Scale bars, 100 and 20 µm. (**e**) High magnification images and single focal planes of whole-mount of E18.5 mesentery and intestine shown in Fig. 1d (after 1 day of lineage tracing). GFP (green) and CD31 (red). Yellow arrowhead marks CD31^{+} GFP^{+} sprouting cell and white arrowheads indicate CD31^{+} GFP^{+} capillaries in the vicinity of the mesenteric artery. Mesentery (Mes), intestine (Int), artery (Ar) and vein (Ve) are indicated. *n* = 6. Scale bars, 200, 100 and 20 µm.

Supplementary Figure 2

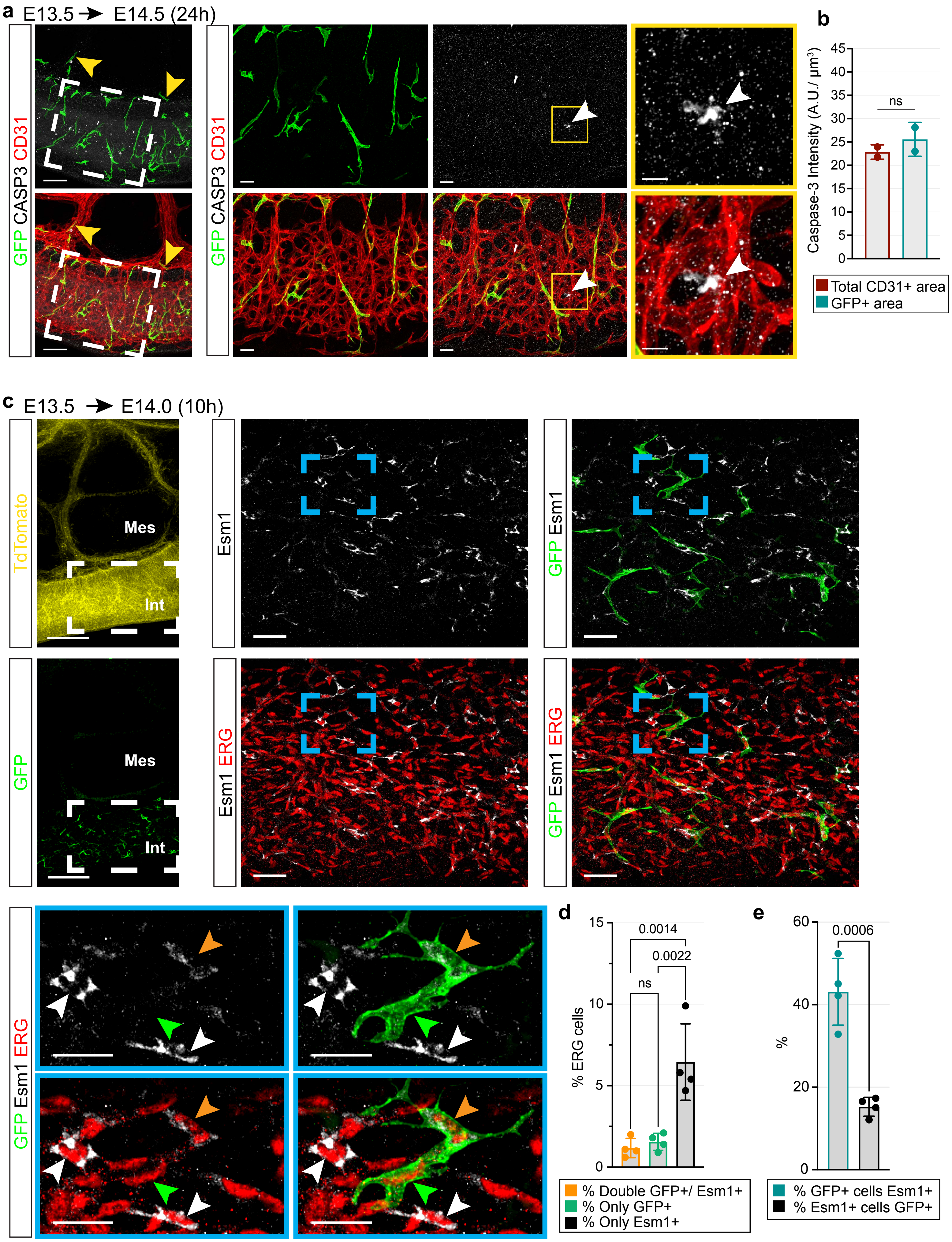

Supplementary Figure 2. *Esm1-CreERT2* activity does not fully respresent total embryonic intestinal Esm1$^+$ ECs.

(a) Whole-mount of E14.5 mesentery and intestine after Esm1 lineage tracing starting at E13.5. GFP (green), CD31 (red) and CASP3 (white). Yellow arrowheads mark GFP$^+$ cells in mesenteric arteries, white arrowhead indicates CASP3$^+$ cell. $n = 2$. Scale bars, 100, 30 and 10 µm. (**b)** Quantification of CASP3 intensity (A.U.) normalized to blood vessel (CD31$^+$) or GFP$^+$ volume (µm^3). **(c)** Whole-mount of E14.0 mesentery and intestine after Esm1 lineage tracing for 10h. GFP (green), ERG (red), Esm1 (white) and Tdtomato (yellow). White arrowheads mark GFP$^-$ Esm1$^+$ cells, orange arrowhead marks GFP$^+$ Esm1$^+$ cell and green arrowhead marks GFP$^+$ Esm1$^-$ cell. Mesentery (Mes) and intestine (Int) are indicated. $n = 4$. Scale bars, 300, 50 and 25 µm. **(d)** Proportion of ERG$^+$ ECs labeled for both GFP$^+$/Esm1$^+$, only GFP$^+$ or only Esm1$^+$. P values, 1-way ANOVA with Tukey post-hoc test; Error bars, Mean ± SD. **(e)** Proportion of GFP$^+$ cells co-staining with Esm1 and proportion of Esm1$^+$ cells co-staining with GFP$^+$. P value, 2-tailed unpaired Student's *t* test; Error bars, Mean ± SD.

Supplementary Figure 3

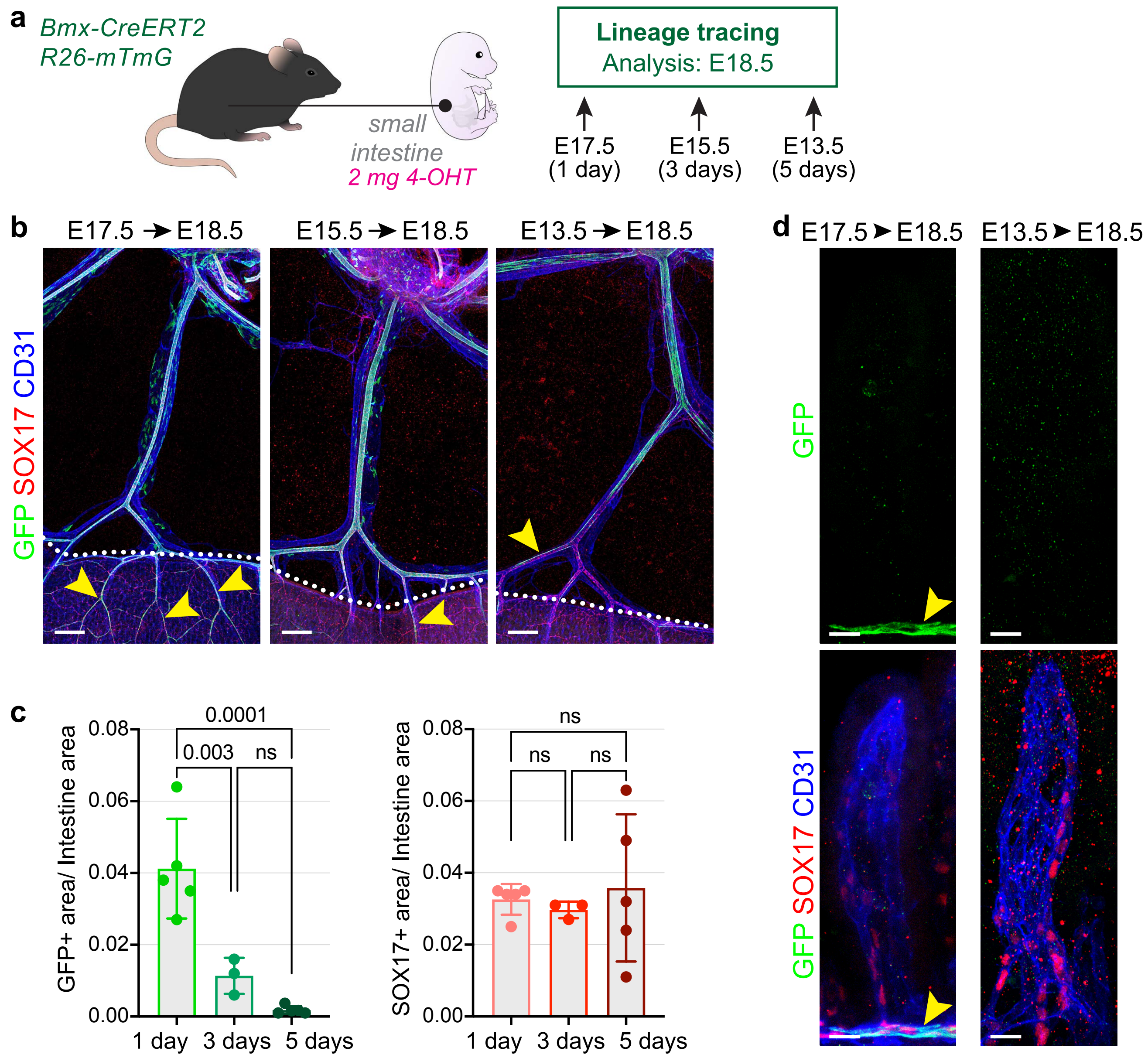

Supplementary Figure 3. *Bmx*-negative ECs contribute to the expansion of large mesenteric arteries.

(**a**) Scheme depicting the experimental design for the tracking of BMX^+ cells. (**b**) *Bmx-CreERT2*-controlled GFP expression (green) predominantly marks large $SOX17^+$ (red) arteries (arrowheads) in E18.5 mesenteries. CD31 (blue). 1 day n = 5; 3 days n = 3; 5 days n = 5. Scale bar, 300 µm. (**c**) Quantification of GFP^+ and $SOX17^+$ area in the intestinal tissue (μm^2, normalized to tissue area). P values, 1-way ANOVA with Tukey post-hoc test (GFP^+ area) and Brown-Forsythe and Welch ANOVA with Dunnett post-hoc test ($SOX17^+$ area); Error bars, Mean ± SD. (**d**) GFP^+ (green) signal is only found in submucosal arteries (arrowhead) after short-term (24 hours) tracking, whereas $SOX17^+$ (red) villus capillaries are devoid of GFP^+ cells in the E18.5 villus. CD31 (blue). 1 day n = 5; 5 days n = 5. Scale bar, 20 µm.

Supplementary Figure 4

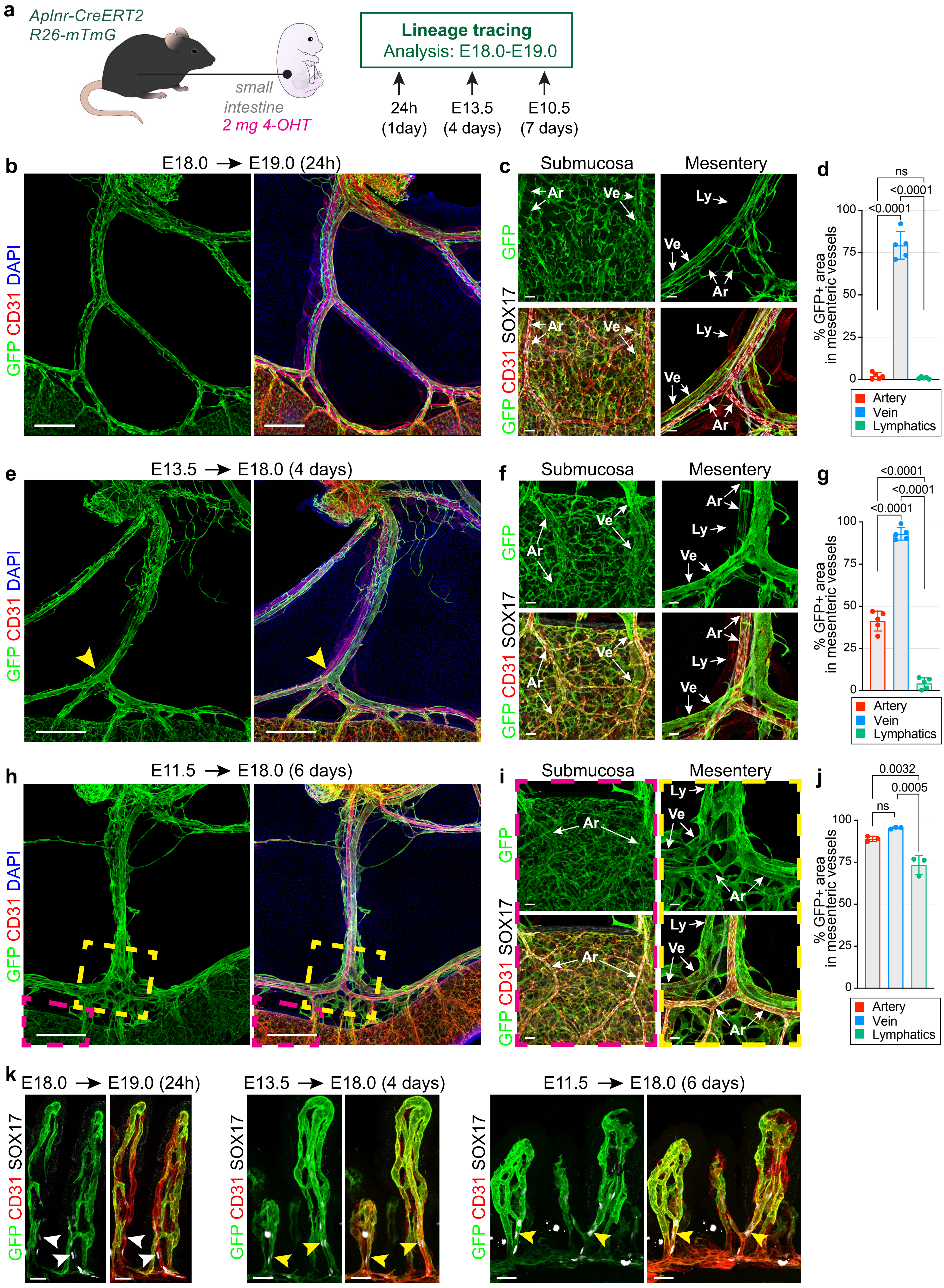

Supplementary Figure 4. Intestinal *Aplnr*-derived ECs generate the mesenteric artery. (**a**) Scheme depicting the experimental design for the tracking of Aplnr$^+$ cells. (**b**, **e**) *Aplnr-CreERT2*-controlled GFP expression (green) predominantly marks large veins and capillaries in E18.0/E19.0 mesenteries 24h after 4-OHT. Aplnr$^+$ cell progeny (green) contributes to the large mesenteric arteries close to the intestine 4 days after 4-OHT. GFP (green), CD31 (red) and DAPI (blue). Yellow arrowhead indicates GFP$^+$ artery. 24h E18.0 n = 4; 24h E19.0 n = 1; 4 days E18.0 n = 5. Scale bar, 500 µm. (**c**, **f**) High magnification images of the intestinal submucosa and mesentery area with SOX17$^+$ (white) arteries. Arrows point at Arteries (Ar), veins (Ve) and lymphatics (Ly). Scale bar, 50 µm. (**d**, **g**) Proportion of GFP$^+$ arteries, veins and lymphatics 24h and 4 days after 4-OHT (µm^2, normalized to vessel area). P values, 1-way ANOVA with Tukey post-hoc test; Error bars, Mean ± SD. (**h**) Whole-mount of E18.0 mesentery and intestine showing that from E11.5, Aplnr$^+$ cell progeny (green) contribute to veins, capillaries and arteries. GFP (green), CD31 (red) and DAPI (blue). E18.0 n = 3. Scale bar, 500 µm. (**i**) High magnification images of the intestinal submucosa and mesentery. Arrows point at GFP signal in capillaries, veins (Ve), lymphatics (Ly) and SOX17$^+$ (white) arteries (Ar). Scale bar, 50 µm. (**j**) Proportion of GFP$^+$ arteries, veins and lymphatics 6 days after 4-OHT (µm^2, normalized to vessel area). P values, 1-way ANOVA with Tukey post-hoc test; Error bars, Mean ± SD. (**k**) In the embryonic intestine, *Aplnr-CreERT2*-labeled (GFP$^+$, green) villus capillaries (CD31, red) are SOX17$^-$ (SOX17, white) 24h after 4-OHT. 4 days and 6 days after 4-OHT most of the villus capillaries are GFP$^+$. White arrowheads, SOX17$^+$ GFP$^-$ villus capillaries. Yellow arrowheads, SOX17$^+$ GFP$^+$ villus capillaries. 24h n = 5; 4 days n = 5; 6 days n = 3. Scale bar, 30 µm.

Supplementary Figure 5

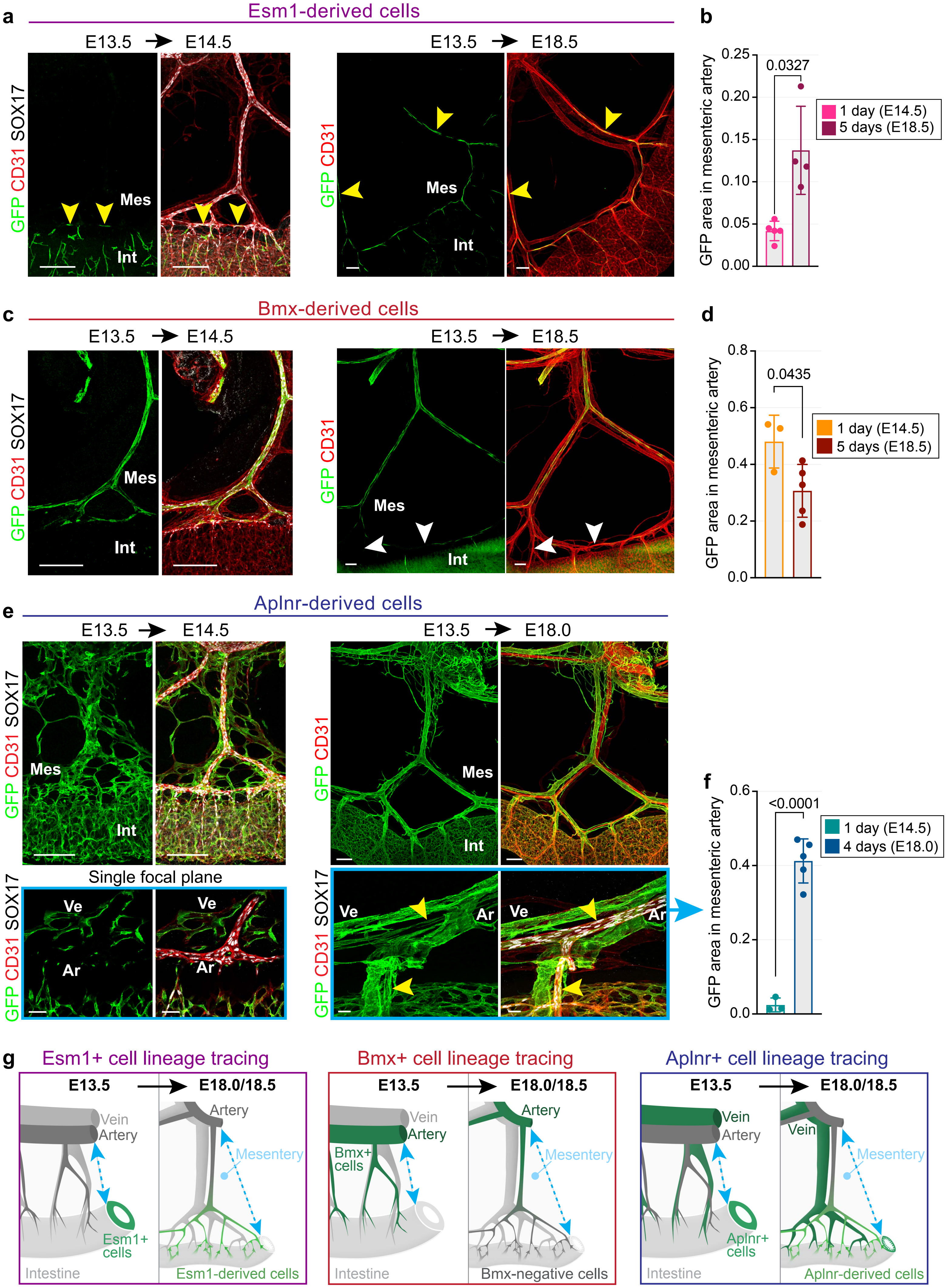

Supplementary Figure 5. Venous-derived intestinal Esm1$^+$ cells give rise to Bmx$^+$ arterial ECs.

(a) Whole-mount of E14.5 and E18.5 mesentery and intestine after Esm1 lineage tracing starting at E13.5. GFP (green), CD31 (red) and SOX17 (white). Yellow arrowheads mark GFP$^+$ cells in mesenteric arteries. Mesentery (Mes) and Intestine (Int) are indicated. Scale bar, 200 μm. **(b)** Proportion of GFP$^+$ mesenteric arteries after 4-OHT at E13.5 (μm^2, normalized to vessel area). P value, Welch's *t* test. Error bars, Mean ± SD. **(c)** Whole-mount of E14.5 and E18.5 mesentery and intestine after Bmx lineage tracing starting at E13.5. GFP (green), CD31 (red) and SOX17 (white). White arrowheads mark CD31$^+$ GFP$^-$ mesenteric arteries close to the intestine. Mesentery (Mes) and Intestine (Int) are indicated. Scale bar, 200 μm. **(d)** Proportion of GFP$^+$ mesenteric arteries after 4-OHT at E13.5 (μm^2, normalized to vessel area). P value, 2-tailed unpaired Student's *t* test. Error bars, Mean ± SD. **(e)** Whole-mount of E14.5 and E18.0 mesentery and intestine after Aplnr lineage tracing starting at E13.5. GFP (green), CD31 (red) and SOX17 (white). Yellow arrowheads mark GFP$^+$ cells in mesenteric arteries. Mesentery (Mes), Intestine (Int), Vein (Ve) and Artery (Ar) are indicated. Scale bar, 200 μm. **(f)** Proportion of GFP$^+$ cells in mesenteric arteries leaving the intestine after 4-OHT at E13.5 (μm^2, normalized to vessel area). The E18.0 time point result is also shown in Supplementary Fig. 4g. P value, 2-tailed unpaired Student's *t* test. Error bars, Mean ± SD. **(g)** Intestinal Esm1-derived and Aplnr-derived cells give rise to Bmx$^+$ arterial ECs in the submucosal and mesenteric arterial network.

Supplementary Figure 6

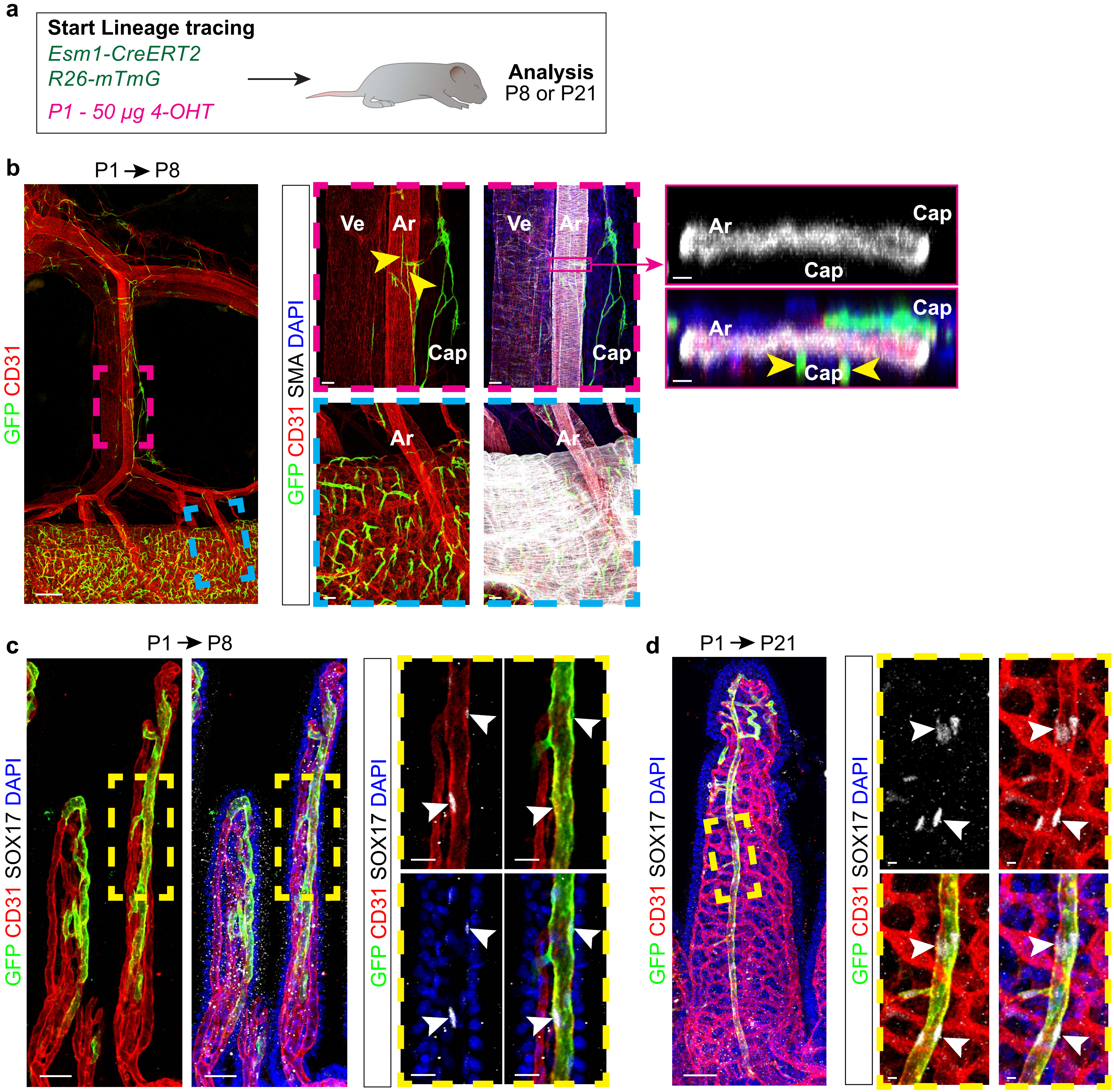

Supplementary Figure 6. *Esm1*-derived ECs make limited postnatal contribution to mesenteric arteries.

(**a**) Experimental design for the tracking of $Esm1^+$ cell progeny after birth. (**b**) Whole-mount of P8 mesentery and intestine with high magnification and frontal view (20 μm depth) showing that GFP signal (green) remains in the intestine and mesenteric capillaries (arrowheads) at 7 days after 4-OHT administration. CD31 (red), SMA (white) and DAPI (blue). Capillary (Cap), artery (Ar) and vein (Ve) are indicated. *n* = 3. Scale bars, 300, 50 and 10 μm. (**c**) GFP^+ ECs remain in the postnatal villus after 7 days of lineage tracing (analysis at P8). GFP (green), CD31 (red), SOX17 (white) and DAPI (blue). Arrows point at GFP^+ $SOX17^+$ ECs. *n* = 4. Scale bars, 40 and 15 μm. (**d**) Whole-mount of P21 intestine showing that *Esm1-CreERT2*-labeled ECs contribute to the $SOX17^+$ arterioles (arrowheads) inside villi after 20 days of lineage tracing. GFP (green), CD31 (red), SOX17 (white) and DAPI (blue). *n* = 3. Scale bars, 50 and 5 μm.

Supplementary Figure 7

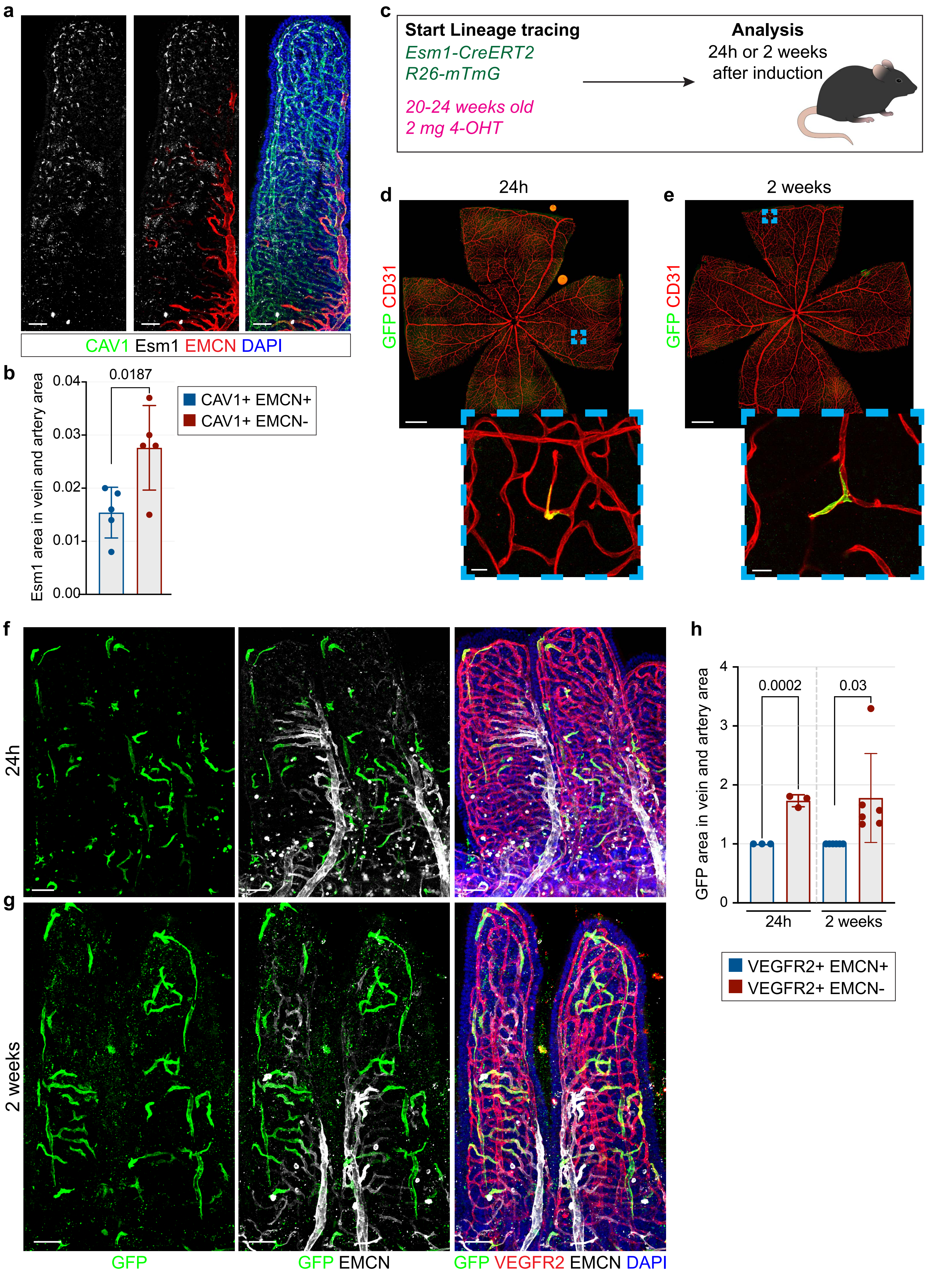

Supplementary Figure 7. Localization of Esm1^{+} cells in the adult villus vasculature.

(**a**) Esm1 (white) is enriched at the apex and the arterial side of the villus vascular network in the duodenum of adult (12-week-old) males. CAV1 (green), EMCN (red) and DAPI (blue). *n* = 5. Scale bar, 50 µm. (**b**) Quantification of the Esm1^{+} area in the venous (CAV1^{+} EMCN^{+}) and arterial (CAV1^{+} EMCN^{-}) villus vasculature (µm^{2}, normalized to vessel area). P values, 2-tailed unpaired Student's *t* test; Error bars, Mean ± SD. (**c**) Experimental design of *Esm1-CreERT2*-mediated cell tracking in adult mice. (**d**, **e**) *Esm1-CreERT2*-labeled (GFP^{+}, green) cells are rare in the adult retinal vasculature (CD31, red) at 24 hours (**d**) and 2 weeks (**e**) after induction. 24 hours *n* = 3, 2 weeks *n* = 5. Scale bars, 500 and 20 µm. (**f**, **g**) Whole-mount of male jejunum showing the distribution of GFP^{+} cells (green) in the mature villus vascular network 24 hours and 2 weeks after 4-OHT administration. VEGFR2 (red), EMCN (white) and DAPI (blue). 24 hours *n* = 3, 2 weeks *n* = 6. Scale bar, 50 µm. (**h**) Graph showing GFP^{+} area (µm^{2}) in the large VEGFR2^{+} EMCN^{-} (µm^{2}, most capillary and arterial) and the smaller VEGFR2^{+} EMCN^{+} (µm^{2}, venous) part of the villus vasculature. Normalization shows that more GFP^{+} cells are found in villus arteries and capillaries compared to veins. P values, 2-tailed unpaired Student's *t* test; Error bars, Mean ± SD.

Supplementary Figure 8

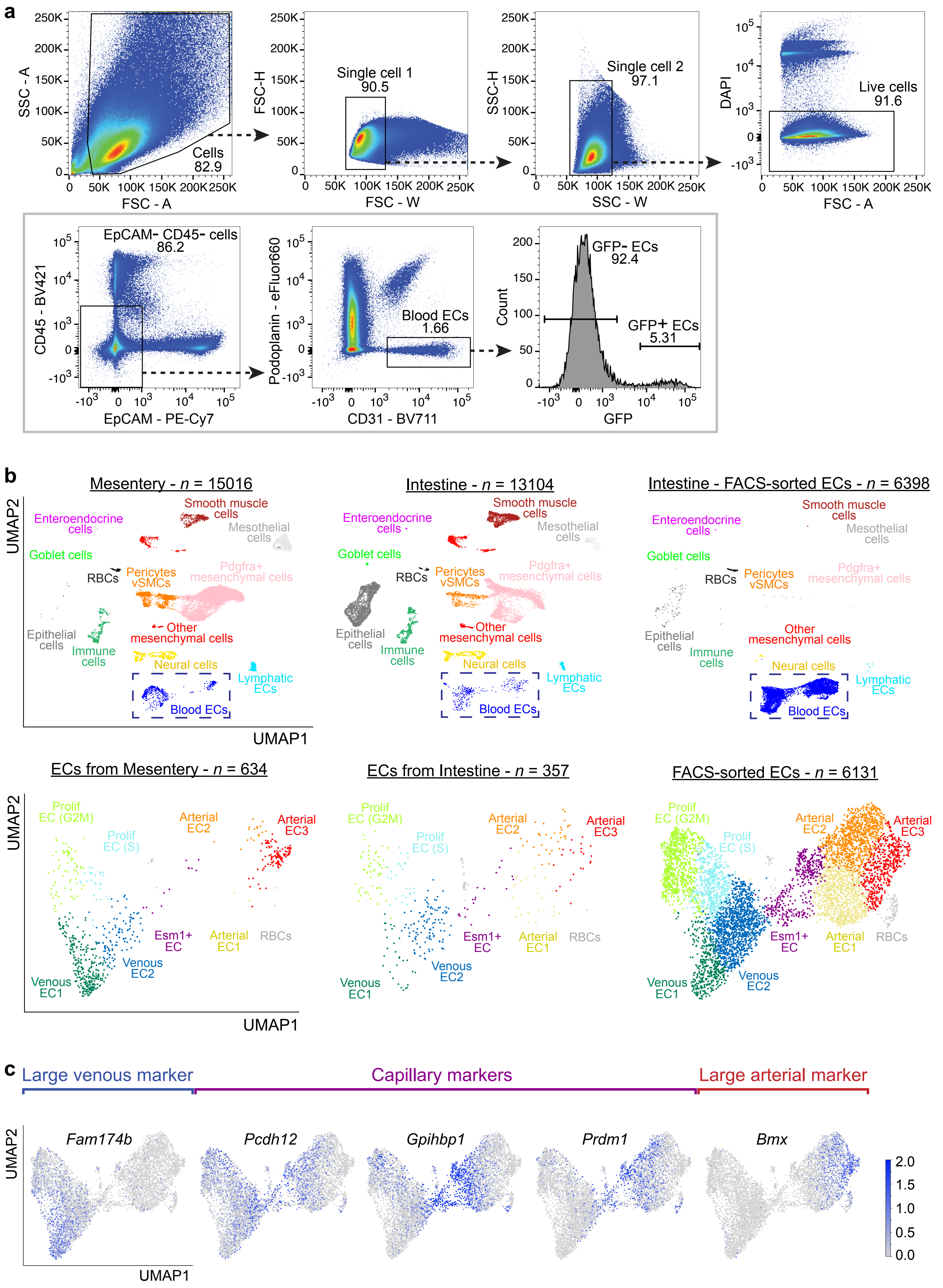

Supplementary Figure 8. EC isolation and scRNA-seq analysis after lineage tracing of Esm1^{+} cells.

(**a**) Flow cytometry gating strategy (CD45^{-} EpCAM^{-} Podoplanin^{-} CD31^{+}) for sorting of GFP^{+} and GFP^{-} ECs from E18.0 embryonic small intestines. (**b**) Individual scRNA-seq UMAP plots of total unsorted mesenteric and small intestinal cells and FACS-sorted GFP^{+} and GFP^{-} blood vessel ECs isolated from E18.0 small intestines. All FACS-sorted GFP^{+} cells were used for scRNA-seq and complemented with GFP^{-} blood vessel ECs. **(c)** UMAP plots showing expression levels of venous (*Fam174b*), arterial (*Bmx*) and capillary markers (*Pcdh12*, *Gpihbp1*, *Prdm1*).

Supplementary Figure 9

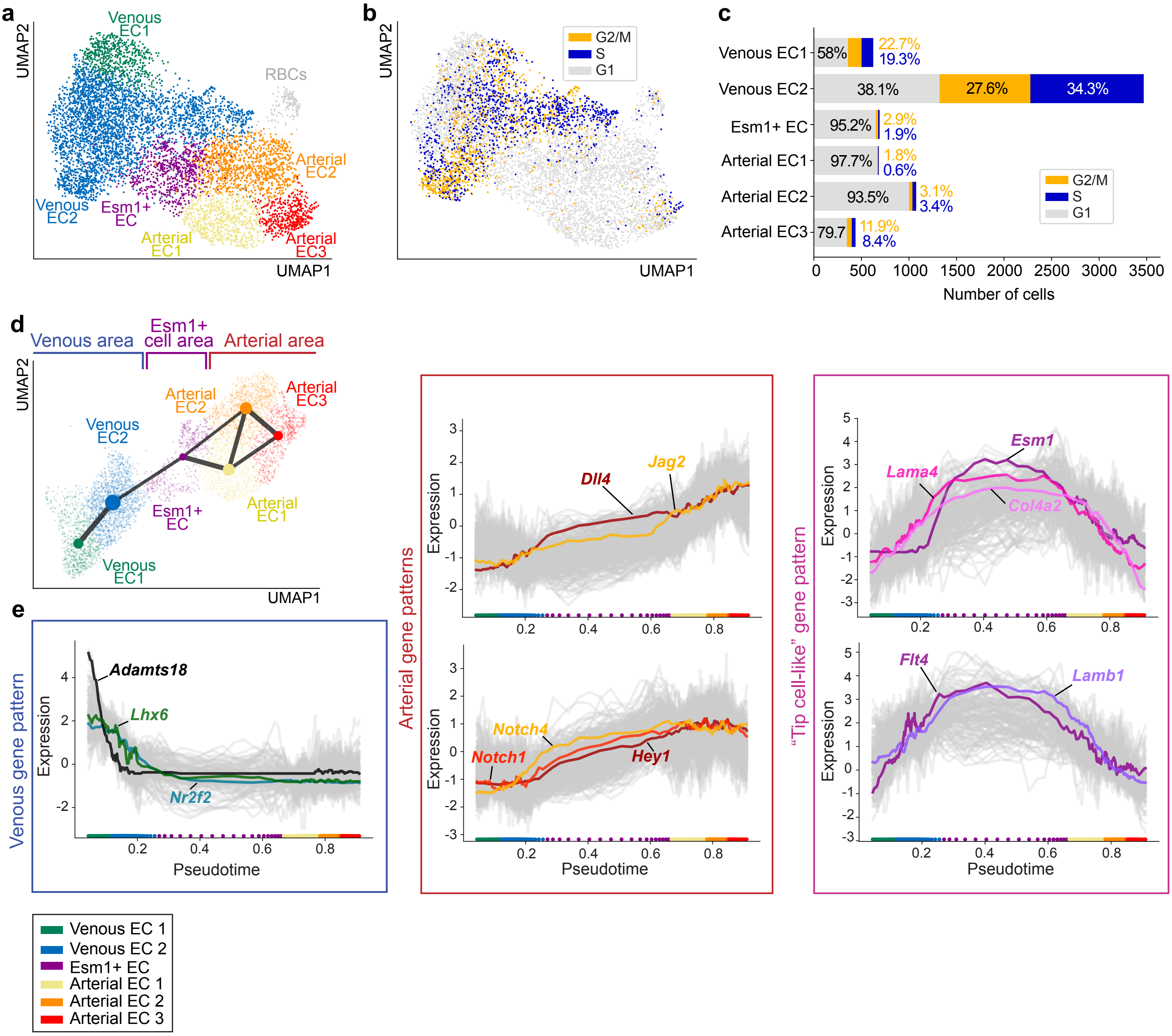

Supplementary Figure 9. Transcriptional zonation in the intestinal vascular network.

(**a**) UMAP plot of all 3 combined scRNA-seq datasets showing blood vessel ECs after cell cycle regression. (**b**) UMAP plot of blood vessel ECs in G2/M, S and G1 phase after cell cycle regression. (**c**) Percentage of cells in G2/M, S and G1 phase in each EC subgroup after cell cycle regression. (**d**) Partition-based graph abstraction (PAGA) analysis of blood vessel ECs (Prolif EC (G2/M) and Prolif EC (S) populations were excluded) showing connectivity between venous and arterial area through Esm1^{+} cell area. Dot size corresponds to the number of cells in each subgroup. (**e**) A total of 22 gene expression patterns were identified. Shown are patterns comprising known venous (*Adamts18*, *Lhx6*, *Nr2f2*), arterial (*Dll4*, *Jag2*, *Notch1*, *Notch4*, *Hey1*) and tip cell-like markers (*Esm1, Lama4*, *Col4a2*, *Flt4, Lamb1*).

Supplementary Figure 10

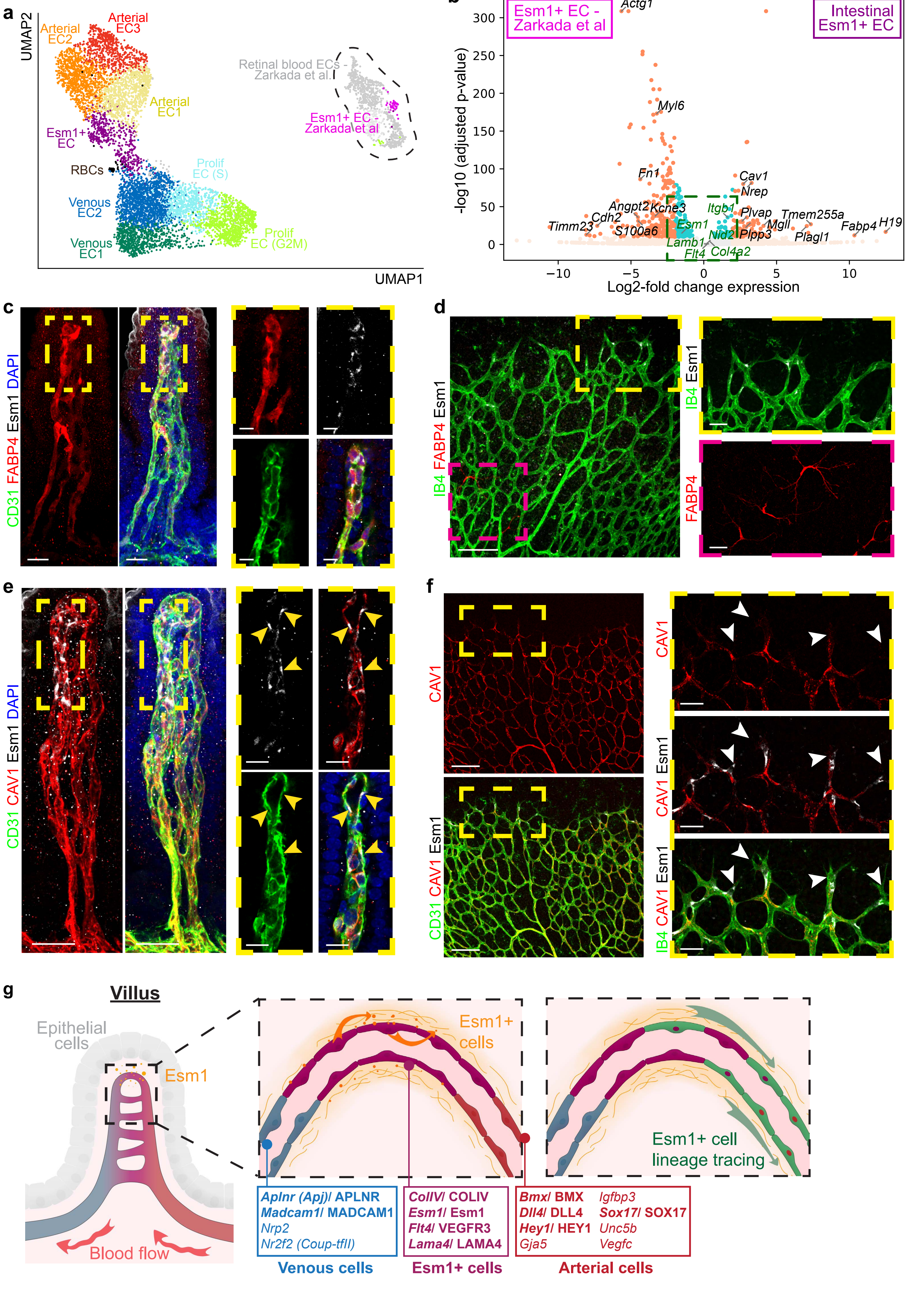

Supplementary Figure 10. Organ-specific blood vessels and Esm1 EC population.

(**a**) UMAP plot of intestinal blood ECs combined with retinal blood EC scRNA-seq datasets from Zarkada et al.[1] (**b**) Volcano plot of differentially expressed genes between Esm1$^+$ ECs from embryonic intestine and P6 wildtype retinal ECs. Genes marked inside green square are not differentially expressed and shared by both retinal and intestinal Esm1$^+$ ECs. (**c**) E18.0 villus vasculature (CD31, green) expresses FABP4 (red). Esm1$^+$ (white) and FABP4$^+$ ECs could be found at the villus apex. n = 3. Sale bars, 20 and 10 µm. (**d**) FABP4 (red) is not expressed in the P5 retinal vasculature (IB4, green). Esm1 (white). n = 4. Scale bars, 100 and 30 µm. (**e**) E18.0 villus vasculature (CD31, green) expresses CAV1 (red). Esm1$^+$ (white) and CAV1$^+$ ECs contribute to the villus capillaries at the villus apex. Yellow arrowheads, CD31$^+$ CAV1$^+$ Esm1$^+$ villus ECs. n = 3. Scale bars, 30 and 15 µm. (**f**) CAV1 (red) is expressed in the P5 retinal vasculature (IB4, green) but downregulated in tip cells (Esm1, white). White arrowheads, IB4$^+$ CAV1^{low} Esm1$^+$ retinal tip cells. n = 4. Scale bars, 100 and 30 µm. (**g**) Diagram depicting vascular zonation in the embryonic intestine and the generation of arterial progenitor cells in the villus apex. Bold text is used for markers validated at the mRNA and protein level.

Supplementary Figure 11

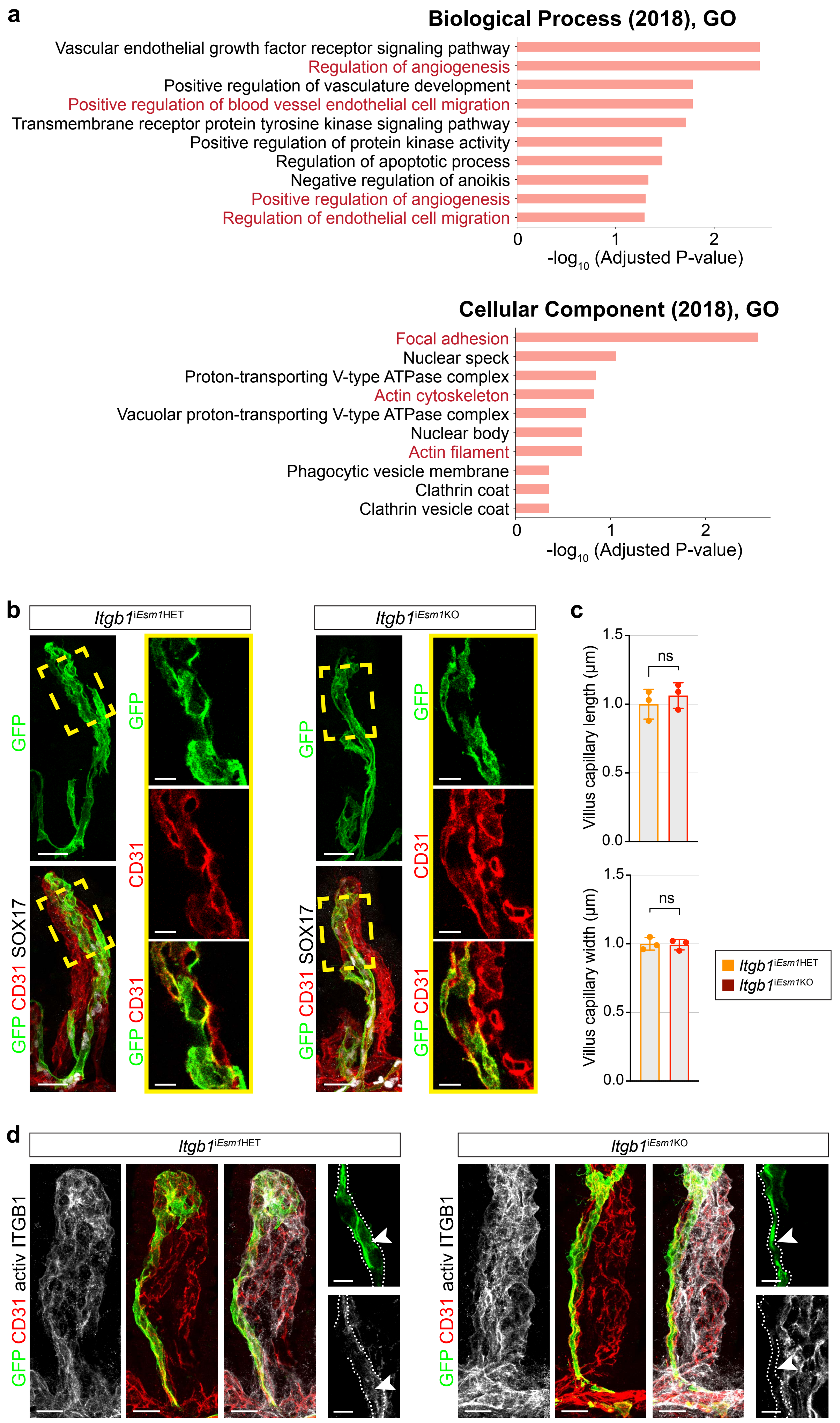

Supplementary Figure 11. Cell migration and focal adhesion genes are enriched in intestinal Esm1$^+$ cells.

(**a**) GO enrichment analysis using genes with expression pattern similar to *Esm1*. **(b)** *Itgb1* loss in Esm1-expressing cells does not impair intestinal villus development. Whole-mount of E18.5 villus with high magnification images stained for GFP (green), CD31 (red) and SOX17 (white). $Itgb1^{iEsm1\mathrm{HET}}$ $n = 3$; $Itgb1^{iEsm1\mathrm{KO}}$ $n = 3$. Scale bars, 30 and 10 µm. (**c**) Quantification of villus capillary length and width (µm). P values, 2-tailed unpaired Student's *t* test; Error bars, Mean ± SD. (**d**) Reduced integrin β1 (ITGB1, white arrowheads) expression in GFP$^+$ (green) ECs (CD31, red) in $Itgb1^{iEsm1\mathrm{KO}}$ E18.5 embryonic intestinal cryosections relative to control. $Itgb1^{iEsm1\mathrm{HET}}$ $n = 3$; $Itgb1^{iEsm1\mathrm{KO}}$ $n = 3$. Scale bars, 20 and 10 µm

Supplementary Figure 12

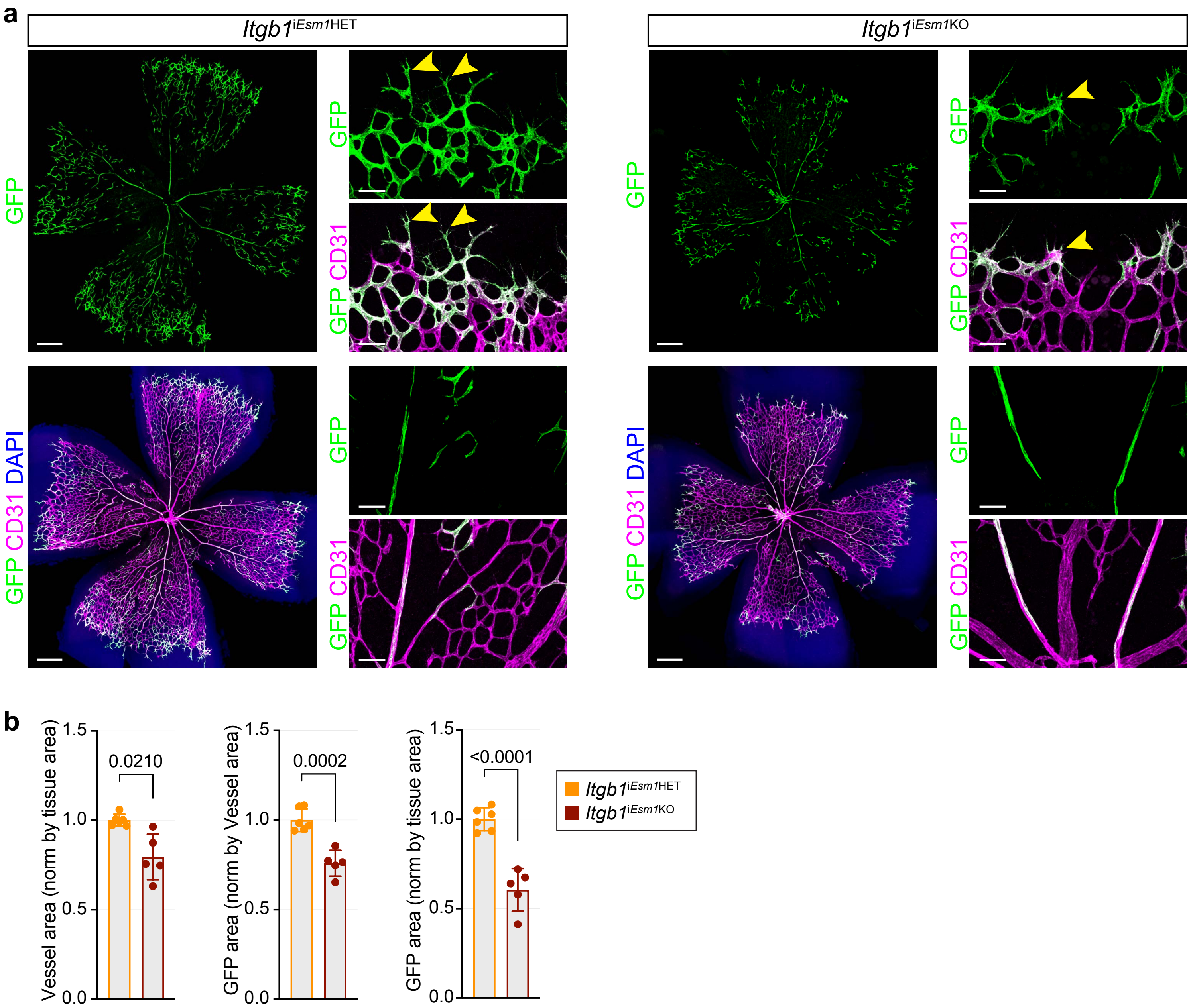

Supplementary Figure 12. Loss of integrin β1 in retinal Esm1^{+} ECs leads to cell pruning and impared sprouting angiogenesis.

(**a**) Whole-mount staining for GFP (green), CD31 (purple) and DAPI (blue) in P6 *Itgb1*$^{\mathrm{i}\mathit{Esm1}\mathrm{KO}}$ retinas shows that *Itgb1* deficiency in Esm1^{+} cells impairs retinal sprouting angiogenesis (yellow arrowheads) relative to control. *Itgb1*$_{\mathrm{i}\mathit{Esm1}\mathrm{HET}}$ *n* = 6, *Itgb1*$_{\mathrm{i}\mathit{Esm1}\mathrm{KO}}$ *n* = 5. Scale bars, 300 and 50 µm. **(b)** Quantification of P6 retina vascular area (in µm^2, CD31 or IB4 area normalized to DAPI area) and GFP^{+} area (in µm^2, GFP^{+} area normalized to vascular (CD31 or IB4) or tissue (DAPI) area) in *Itgb1*$^{\mathrm{i}\mathit{Esm1}\mathrm{HET}}$ and *Itgb1*$^{\mathrm{i}\mathit{Esm1}\mathrm{KO}}$ mutant littermates. *Itgb1*$^{\mathrm{i}\mathit{Esm1}\mathrm{HET}}$ group set to 1. P values, Welch's *t* test (vessel area) and 2-tailed unpaired Student's *t* test (GFP^{+} area); Error bars, Mean ± SD.

Supplementary Figure 13

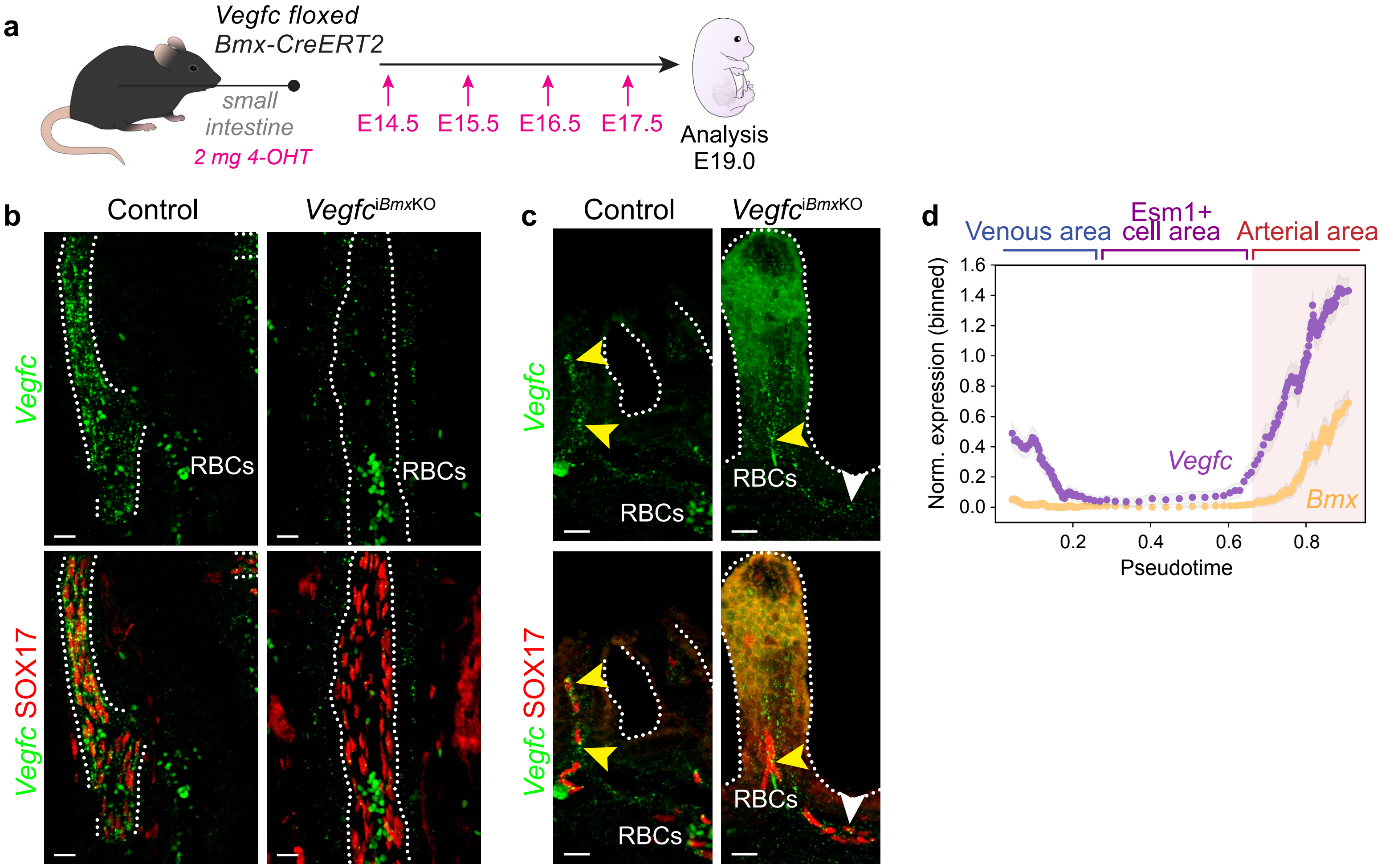

Supplementary Figure 13. *Bmx*-controlled loss of *Vegfc* in mesenteric and intestinal arteries.

(**a**) Experimental scheme for *Vegfc* loss-of-function experiments in Bmx^{+} arterial ECs. (**b**) RNAscope analysis of cryosection from E19.0 mesenteries showing that *Vegfc* (green) is strongly expressed in large mesenteric arteries (SOX17, red) and is lost upon *Bmx-CreERT2*-controlled inactivation. Note unspecific staining of red blood cells (RBCs). 5 Control and 3 $Vegfc^{iBmx\mathrm{KO}}$ samples were analyzed. Scale bar, 15 µm. (**c**) Cryosections of E19.0 intestines showing that *Vegfc* (green) is expressed by the $SOX17^{+}$ (red) villus ECs. Yellow arrowheads indicate $Vegfc^{+}$ $SOX17^{+}$ villus capillaries, white arrowhead $Vegfc^{low}$ $SOX17^{+}$ submucosal arteries in $Vegfc^{iBmx\mathrm{KO}}$ samples. 5 Control and 3 $Vegfc^{iBmx\mathrm{KO}}$ samples. Scale bar, 15 µm. (**d**) Pseudotime plot showing increased *Vegfc* expression at the interphase between the $Esm1^{+}$ and arterial region, preceeding *Bmx* upregulation.

Supplementary Figure 14

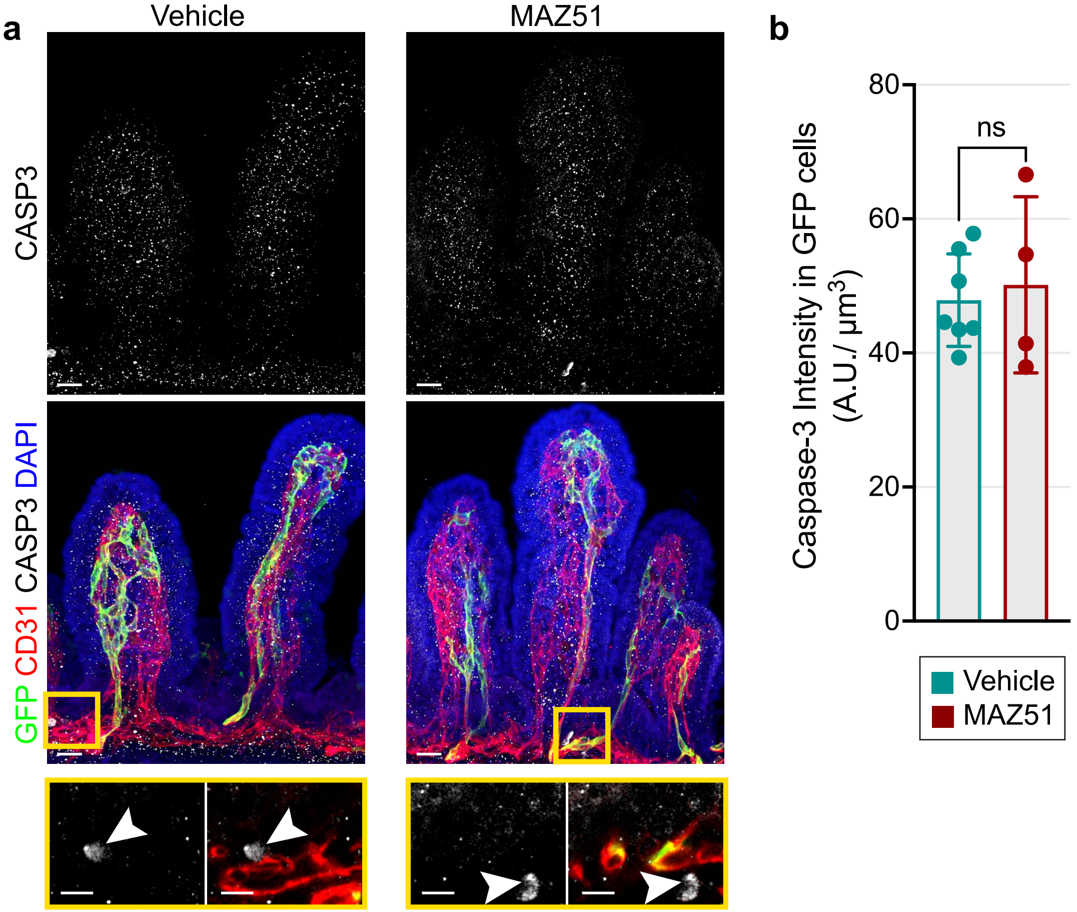

Supplementary Figure 14. MAZ51 treatment does not induce intestinal EC apoptosis.

(a) Whole-mount of E18.0 intestine 48h after *Esm1-CreERT2*-mediated reporter activation during vehicle or MAZ51 treatment. GFP (green), CD31 (red), CASP3 (white) and DAPI (blue). Arrowheads mark $CASP3^+$ cells. Vehicle n = 7; MAZ51 n = 4. Scale bars, 20 and 10 µm. **(b)** Quantification of CASP3 intensity (A.U.) normalized to GFP^+ volume (μm^3). P values, 2-tailed unpaired Student's t test; Error bars, Mean ± SD.

Supplementary Figure 15

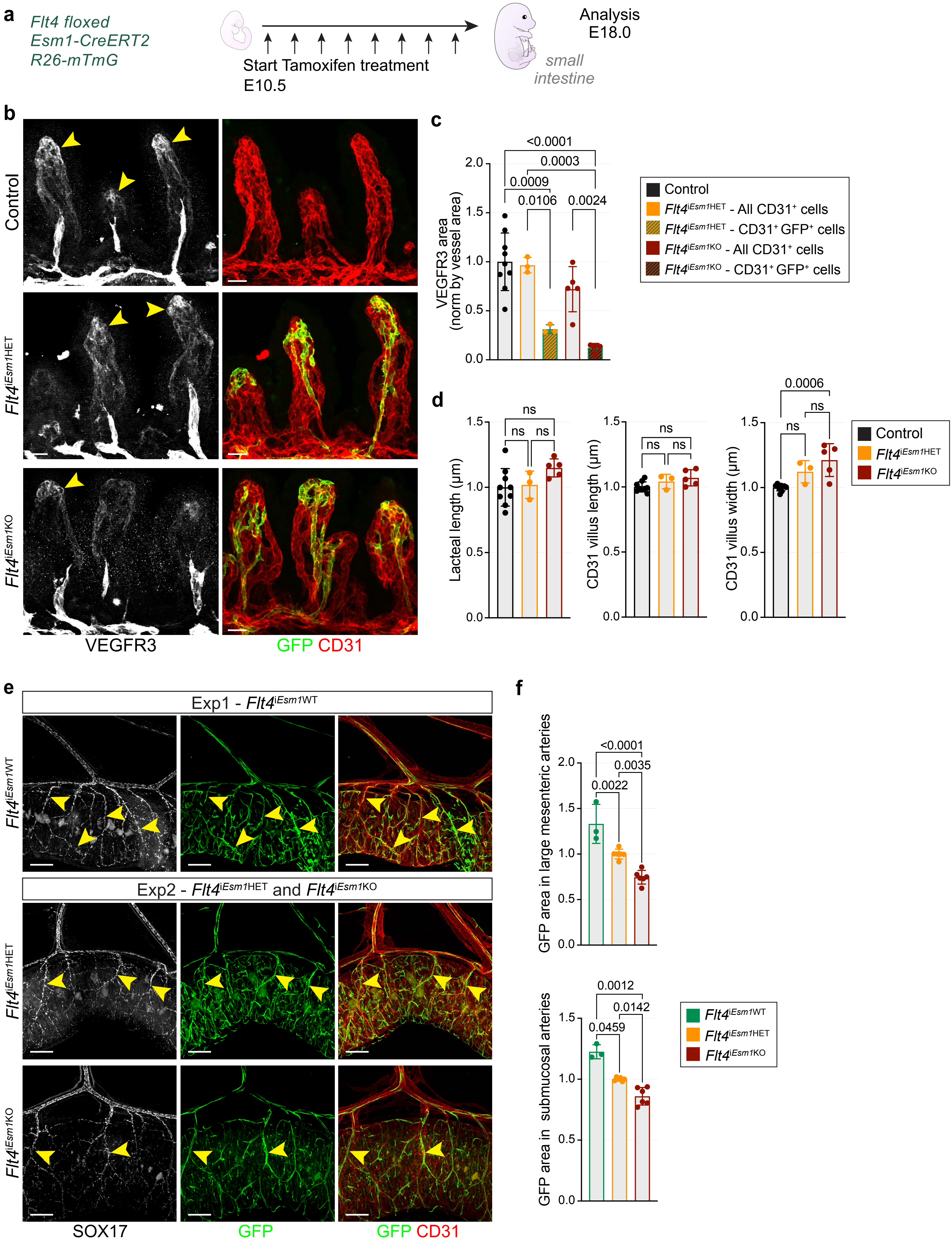

Supplementary Figure 15. Loss of *Flt4* reduces Esm1^{+} cell contribution to large mesenteric arteries.

(**a**) Experimental design for Esm1^{+} cell-specific *Flt4* inactivation from E10.5. (**b**) Gradual loss of VEGFR3 (white) expression and increased villus capillary width in *Flt4*$^{iEsm1\text{HET}}$ heterozygous and *Flt4*$^{iEsm1\text{KO}}$ homozygous mutant villi compared to control littermates at E18.0. GFP (green) and CD31 (red). Arrowheads mark VEGFR3^{+} capillaries in villus apex. Control n = 9; *Flt4*$^{iEsm1\text{HET}}$ n = 3 and *Flt4*$^{iEsm1\text{KO}}$ n = 5. Scale bar, 30 µm. (**c**) Quantification of VEGFR3^{+} area in CD31^{+} and CD31^{+} GFP^{+} villus blood vessels (in µm^{2}, normalized to CD31 area). P values, 1-way ANOVA with Tukey post-hoc test; Error bars, Mean ± SD. (**d**) Quantification of VEGFR3 villus lacteal length (µm), villus capillary network length and width (µm). P values, 1-way ANOVA with Tukey post-hoc test; Error bars, Mean ± SD. (**e**) Whole-mount of E18.0 intestine and mesentery showing reduced GFP^{+} cell (green, arrowheads) contribution to the intestinal submucosal and mesenteric arteries (CD31, red; SOX17, white) in *Flt4*$^{iEsm1\text{KO}}$ mutants compared to *Flt4*$^{iEsm1\text{WT}}$ and *Flt4*$^{iEsm1\text{HET}}$ animals. *Flt4*$^{iEsm1\text{WT}}$ n = 3 ; *Flt4*$^{iEsm1\text{HET}}$ n = 6 and *Flt4*$^{iEsm1\text{KO}}$ n = 6. Scale bar, 200 µm. (**f**) GFP area in submucosal and mesenteric arteries (in µm^{2}, normalized to artery area). 4 experiments were pooled and *Flt4*$^{iEsm1\text{HET}}$ normalized to 1. P values, 1-way ANOVA with Tukey post-hoc test (mesentery) and Brown-Forsythe and Welch ANOVA with Dunnett post-hoc test (submucosa); Error bars, Mean ± SD.

Supplementary Table 1. Primary and secondary antibodies and dyes used for staining

Antigen	Reactivity	Species	Source	Dilution
**Primary antibodies**				
Cleaved Caspase-3	Human/Mouse/Rat	Rabbit	Cell Signaling (#9661)	1:100
Caveolin-1	Human/Mouse/Rat	Rabbit	Cell Signaling (#3238)	1:100
CD31	Human/Mouse/Rat	Goat	R&D Systems (#AF3628)	1:200
CD31	Human/Mouse	Rabbit	Abcam (#ab28364)	1:100
CD31	Mouse	Rat	BD Pharmingen (#553370)	1:100
Collagen IV	Mouse	Rabbit	Chemicon (#AB756P)	1:500
Dll4	Mouse	Goat	R&D (#AF1389)	1:50
E-cadherin	Human/Mouse	Rabbit	Cell Signaling (#3195)	1:200
Endomucin	Mouse	Rat	Santa Cruz (#SC-65495)	1:100
Erg	Human/Mouse	Rabbit	Abcam (#ab110639)	1:100
Esm1	Mouse	Goat	R&D (#AF1999)	1:100-200
Fabp4	Human/Mouse	Rabbit	Abcam (#ab13979)	1:200
GFP	-	Chicken	2BScientific Ltd (#GFP-1010)	1:1000
IB4 - Biotinylated	Mouse/Rat/ Rabbit/Goat	Griffonia simplicifolia	Vector (#B-1205)	1:25
Integrin b1	Mouse	Rat	BD Pharmingen (#553715)	1:200
Laminin α4	Mouse	Rabbit	Serum 377 (Gift from L. Sorokin)[2]	1:400
Lyve1	Mouse	Rat	R&D (#MAB2125)	1:500
MAdCAM1	Mouse	Rat	Abcam (#ab80680)	1:200
Prox1	Human	Rabbit	ReliaTech (#102-PA32AG)	1:100
αSMA - Cy3	Human/Mouse/Rat	Mouse	Sigma (#C6198)	1:500
αSMA - eFluor660	Human/Mouse/Rat	Mouse	eBioscience (#50-9760-82)	1:100
Sox17	Human	Goat	R&D (#AF1924)	1:100
Vegfr2	Mouse	Goat	R&D (#AF644)	1:100
Vegfr3	Mouse	Goat	R&D (#AF743)	1:100

**Secondary antibodies**				
Alexa 405-conjugated Streptavidin	-	-	Invitrogen (#S32351)	1:100
Alexa 488–conjugated	Chicken	Donkey	Jackson Laboratories (#703-545-155)	1:300
Alexa 488–conjugated	Goat/Rabbit/ Rat	Donkey	Invitrogen (#A11055, #A21206, #A21208)	1:300
Alexa 594–conjugated	Goat/Rabbit/ Rat	Donkey	Invitrogen (#A11058, #A21207, #A21209)	1:300
Alexa 647–conjugated	Goat/Rabbit	Donkey	Invitrogen (#A21447, #A31573)	1:300
Alexa 647–conjugated	Rat	Donkey	Jackson ImmunoResearch (#712-605-153)	1:300
**Dyes**				
DAPI	-	-	Sigma (#D9542)	
Click-It EdU Alexa Fluor 647	-	-	Invitrogen (#C10340)	

Supplementary Table 2. RNAscope reagents

Reagents	Source
RNAscope Multiplex Fluorescent Reagent Kit v2	ACD (#323100)
RNA-Protein Co-Detection Ancillary Kit	ACD (#323180)
RNAscope 3-plex Positive Control Probe - Mm	ACD (#320881)
RNAscope 3-plex Negative Control Probe - Mm	ACD (#320871)
RNAscope Probe - Mm - Vegfc	ACD (#492701)
TSA Plus Fluorescein	Akoya Biosciences (#NEL741001KT)

Supplementary Table 3. FACS-sorting antibodies

Antigen	Reactivity	Specie	Source	Dilution
**FACS antibodies**				
CD31-BV711	Mouse	Rat	BD Biosciences (#740680)	1:200
CD45- BV421	Mouse	Rat	Biolegend (#103134)	1:200
Epcam - PE-Cy7	Mouse	Rat	BioLegend (#118216)	1:50
Podoplanin - eFluor660	Mouse	Hamster	eBioscience (#50-5381)	1:200

References

1. Zarkada, G. *et al.* Specialized endothelial tip cells guide neuroretina vascularization and blood-retina-barrier formation. *Dev Cell* **56**, 2237-2251.e6 (2021).

2. Ringelmann, B. *et al.* Expression of Laminin α1, α2, α4, and α5 Chains, Fibronectin, and Tenascin-C in Skeletal Muscle of Dystrophic 129ReJ*dy*/*dy*Mice. *Experimental Cell Research* **246**, 165–182 (1999).
